# Effects of *Rhodiola rosea* and caffeine, alone and in combination, on Wingate performance and physiological stress responses in soccer players

**DOI:** 10.3389/fphys.2026.1964904

**Published:** 2026-10-19

**Authors:** Mehmet Emin Yelken, Sezgin Hepsert, Hakan Küçüksayan, Engin Vural, Sedat Gülten, Mehmet Akif Bildirici, Neslihan Akçay

**Affiliations:** 1 Provincial Directorate of Youth and Sports, Ministry of Youth and Sports, Cankırı, Türkiye; 2 Ministry of National Education, Elazığ, Türkiye; 3 Department of Medical Biochemistry, Kastamonu University, Kastamonu, Türkiye; 4 Ministry of National Education, Bitlis, Türkiye; 5 Hasan Doğan Faculty of Sport Sciences, Karabuk University, Karabuk, Türkiye

**Keywords:** alpha amylase, anaerobic performance, caffeine, cortisol, ergogenic aids, *Rhodiola rosea*, Wingate test

## Abstract

**Background:**

Caffeine is a well-established ergogenic aid that enhances physical performance and modulates central nervous system activity. *Rhodiola rosea* (RR) has gained attention for its potential anti-fatigue and stress-regulating effects; however, evidence on its combined use with caffeine remains limited.

**Objective:**

This study aimed to investigate the acute effects of caffeine, RR, and their combination on anaerobic performance and salivary stress biomarkers in trained male soccer players.

**Methods:**

Ten healthy male soccer players (age: 20.20 ± 1.54 years) participated in a randomized, crossover, placebo-controlled design. Participants completed four conditions in a randomized order: placebo (P), caffeine (C, 3 mg·kg^-1^), RR (3 mg·kg^-1^), and combined caffeine + RR (CRR, 3 mg·kg^-1^ each). Sixty minutes after supplementation, participants performed the Wingate Anaerobic Test. Salivary cortisol, α-amylase, and immunoglobulin A (IgA) were analyzed using ELISA. Data were assessed using repeated-measures ANOVA with *post hoc* tests.

**Results:**

Significant differences were observed across experimental conditions. The CRR condition showed higher maximum power output compared with the placebo condition (p<0.05), whereas average power output was higher in the CRR condition than in the RR condition (p<0.05). Cortisol levels differed significantly between the CRR and placebo conditions (p<0.05). α-Amylase levels were significantly lower in the caffeine condition than in the placebo condition (p<0.05). IgA levels differed significantly between the RR and placebo conditions (p<0.05).

**Conclusion:**

Acute caffeine and Rhodiola rosea supplementation, alone or in combination, was associated with differences in anaerobic performance and salivary biomarkers across experimental conditions. Given the small sample size and acute supplementation design, these findings should be interpreted cautiously. Further research with larger samples and appropriate interaction analyses is warranted to clarify the independent and combined effects of caffeine and Rhodiola rosea.

## 1 Introduction

Soccer is a team sport characterized by repeated sprints, sudden changes in direction, and explosive power, requiring a high level of aerobic and anaerobic capacity (Bangsbo et al., 2006). During the game, soccer players are exposed to short-term maximal power output and physiological and psychological stress parameters, which have a decisive impact on their performance (Mohr et al., 2003). The use of supplements for high-level sports performance has become quite popular today. Effective natural methods are being sought to counteract the adverse physiological and psychological effects of intense training programs, such as fatigue, decreased performance, and metabolic stress (Hepsert et al., 2026). Consequently, the use of natural herbal supplements has emerged as an important area of research. In this context, caffeine and RR emerge as important research topics due to their potential positive effects on performance loss and stress caused by overtraining (Maughan et al., 2018; Yun et al., 2024).

RR is a plant species native to North America, Asia, and certain regions of Europe, and it has garnered attention particularly for its fatigue-reducing and performance-enhancing effects (Wang et al., 2025a). The literature indicates that it is a potential ergogenic aid that can improve both physical and mental performance parameters in athletes due to its anti-hypoxic and anti-fatigue properties (Wang et al., 2025b). The plant’s rich phytochemical composition enables it to exhibit adaptogenic properties. Thus, it is reported to enhance the body’s overall resilience, strengthen resistance to stress, and positively influence exercise-induced adaptation processes (Tinsley et al., 2024). Additionally, findings regarding the effects of RR supplementation on performance are not entirely consistent. It has been reported that acute use provides significant ergogenic effects primarily on physical performance rather than on visual-cognitive or mental outcomes (Marcos-Frutos et al., 2025). It has also been suggested that RR may improve endurance performance by reducing perceived exertion (Noreen et al., 2013). While some studies have shown that this plant does not elicit significant performance gains in isolation (Duncan et al., 2016), its unique adaptogenic properties present a strong physiological rationale for co-administration with other central nervous system stimulants, potentially maximizing ergogenic outcomes during high-intensity stress.

Caffeine is one of the most widely consumed psychoactive substances worldwide and is frequently used to enhance athletic performance (Goldstein et al., 2010). Its ergogenic effects primarily arise through the antagonism of adenosine receptors, reducing the perception of fatigue and exertion while increasing central nervous system stimulation. Concurrently, improvements are observed in motor control, attention, and alertness levels (Spriet, 2014; Okut and Sarı, 2025). The literature suggests that caffeine consumption has positive effects on endurance performance, reaction time, and concentration, and provides significant advantages, particularly in sports disciplines that require endurance and sustained attention (Grgic et al., 2018). When considering the complementary mechanisms of these two substances, combining the acute central nervous system stimulation of caffeine with the cellular stress-modulating and anti-fatigue properties of RR could theoretically provide a dual-action ergogenic benefit. Specifically, this combination may concurrently optimize mechanical power output and attenuate the acute neuroendocrine stress responses typically induced by exhaustive anaerobic bouts, such as intermittent soccer-specific workloads (Duncan et al., 2016; Grgic et al., 2018).

Despite the growing body of evidence supporting the ergogenic effects of caffeine and RR, several important gaps remain in the literature. Although previous studies have demonstrated beneficial effects of these supplements when administered individually, evidence regarding their combined administration remains limited. Recent crossover studies have also highlighted the growing interest in combined ergogenic interventions; however, most have evaluated caffeine in combination with other nutritional or behavioral strategies rather than with adaptogenic herbal supplements such as *RR* (Arslan et al., 2025; Arslan et al., 2026). Furthermore, previous investigations have primarily focused on performance outcomes, whereas studies simultaneously evaluating physiological stress biomarkers together with anaerobic performance are scarce. In addition, evidence derived specifically from trained soccer players performing the Wingate anaerobic test remains limited. Therefore, it remains unclear whether the acute administration of caffeine and RR, alone or in combination, produces different effects on anaerobic performance and exercise-related salivary biomarkers when directly compared within the same experimental design (Wang et al., 2025; Saavedra-Velásquez et al., 2024).

In this context, the present study was designed to address this gap by directly comparing the acute effects of placebo, caffeine, RR, and combined caffeine + RR supplementation in a randomized crossover design. The study evaluated Wingate anaerobic performance, a widely accepted and validated laboratory measure of anaerobic power and capacity (Beneke et al., 2002), together with salivary biomarkers to characterize performance and acute physiological responses across the four experimental conditions. Although soccer requires a robust aerobic foundation for the duration of matches, its most decisive actions, such as sprinting, tackling, and rapid transitions, are predominantly powered by anaerobic pathways. Therefore, the Wingate protocol provides a standardized laboratory-based measure of anaerobic power that is relevant to high-intensity actions in soccer players. Salivary cortisol, α-amylase, and immunoglobulin A (IgA) were included because they provide complementary information regarding neuroendocrine stress, sympathetic nervous system activation, and mucosal immune function following high-intensity exercise. The simultaneous assessment of these biomarkers and Wingate performance provides complementary information on performance and acute physiological responses following the different supplementation conditions. However, these biomarkers do not directly establish the underlying mechanisms of any observed performance effects (Kreipke et al., 2021). By integrating performance and biomarker responses, this study seeks to provide a more holistic perspective on supplementation strategies. The findings are expected to contribute to the development of evidence-based nutritional and supplementation approaches to optimize athletic performance. Based on the previously reported effects of caffeine and RR, we hypothesized that supplementation condition would influence Wingate anaerobic performance and salivary biomarkers, with differences expected among the placebo, caffeine, RR, and combined caffeine + RR conditions.

## 2 Materials and methods

### 2.1 Participants

The sample size was calculated using G*Power software (Heinrich Heine University Düsseldorf, Düsseldorf, Germany) using the F-test family (ANOVA: repeated measures, within factors), assuming four experimental conditions, an effect size (f) of 0.40, a Type I error rate (α) of 0.05, a statistical power (1−β) of 0.80, a correlation of 0.50 among repeated measures, and a nonsphericity correction (ϵ) of 1.00. The effect size of f = 0.40 was specified *a priori* as the anticipated effect magnitude for the primary performance outcomes and was considered a relatively large effect according to conventional interpretations of Cohen’s f. This value was not derived from a single previous study. Importantly, ϵ = 1.00 represented the nonsphericity correction factor rather than the assumed effect size. No separate *a priori* power calculations were performed for the individual salivary biomarker outcomes; therefore, the sample size was determined based on the overall repeated-measures study design. Ten competitive amateur male soccer players participated in the study (age: 20.20 ± 1.54 years; height: 179.50 ± 5.66 cm; body weight: 68.84 ± 8.77 kg; BMI: 21.48 ± 2.34 kg/m²). All participants trained regularly and were considered clinically healthy based on a self-reported health questionnaire and detailed medical history assessment. All participants were licensed soccer players who had been actively engaged in soccer for the previous two years and participated in regular soccer training three days per week. Habitual daily caffeine intake was not quantitatively assessed before the study. However, participants were instructed to abstain from coffee, caffeine-containing products, and other caffeine sources for 72 h before each experimental session. The inclusion criteria were: (i) being a competitive amateur male soccer player; (ii) having no diagnosed chronic disease; (iii) being a non-smoker and not consuming alcohol regularly; (iv) not using pharmacological experimental conditions, prescription medications, or ergogenic supplements during the previous three months; and (v) having no known hypersensitivity to caffeine or RR. Participants were excluded if they had a known allergy or intolerance to any substance used in the study; cardiovascular, metabolic, neurological, psychiatric, or other systemic chronic diseases; a serious musculoskeletal injury within the previous six months; current use of performance-enhancing supplements; or extreme or restrictive dietary practices that could substantially affect energy balance. Before participation, all participants were informed about the study’s purpose, procedures, and potential risks and provided written informed consent. The study was conducted in accordance with the Declaration of Helsinki and was approved by the Institutional Review Board of the Bingöl University Health Sciences Scientific Research and Publication Ethics Committee (November 20, 2025; Meeting No. 11, Decision No. 1).

### 2.2 Study design

The study was conducted using a single-blind, randomized, four-condition crossover design. Each participant completed all four experimental conditions in a randomized sequence: placebo (P), caffeine (C), Rhodiola rosea (RR), and combined caffeine and Rhodiola rosea (CRR). The order of the experimental conditions was generated using the randomization function of Random.org (Randomness and Integrity Services Ltd., Dublin, Ireland), and each participant was assigned a randomly generated sequence of the four experimental conditions. Participants were blinded to the supplementation condition, whereas the investigator responsible for supplement administration was aware of the assigned condition. Before the experimental sessions, all participants completed a familiarization session to become accustomed to the experimental procedures, equipment, and testing protocol. The familiarization session included a standardized warm-up and a practice Wingate anaerobic test. A 72-h washout period was implemented between consecutive experimental sessions to minimize potential carry-over effects (Thomas et al., 2023). Each experimental session followed the same standardized protocol. At the beginning of each session, the assigned supplement was administered according to the assigned experimental condition. Participants then rested for 60 min to allow the administered substances to be absorbed. Following this period, participants completed a standardized warm-up and performed a 30-s Wingate anaerobic test. Thus, a Wingate anaerobic test was performed after each of the four experimental supplementation conditions. Immediately following the Wingate test, saliva samples were collected for subsequent biochemical analysis. Salivary cortisol, α-amylase, and immunoglobulin A (IgA) concentrations were determined using enzyme-linked immunosorbent assay (ELISA) methods. All experimental procedures, including supplementation, the 60-min post-supplementation period, warm-up, Wingate anaerobic testing, saliva collection, and biochemical analyses, were performed according to the same standardized protocol across all four experimental conditions. The experimental sequence and overall study procedure are presented in **Figure 1**.

**Figure 1:**
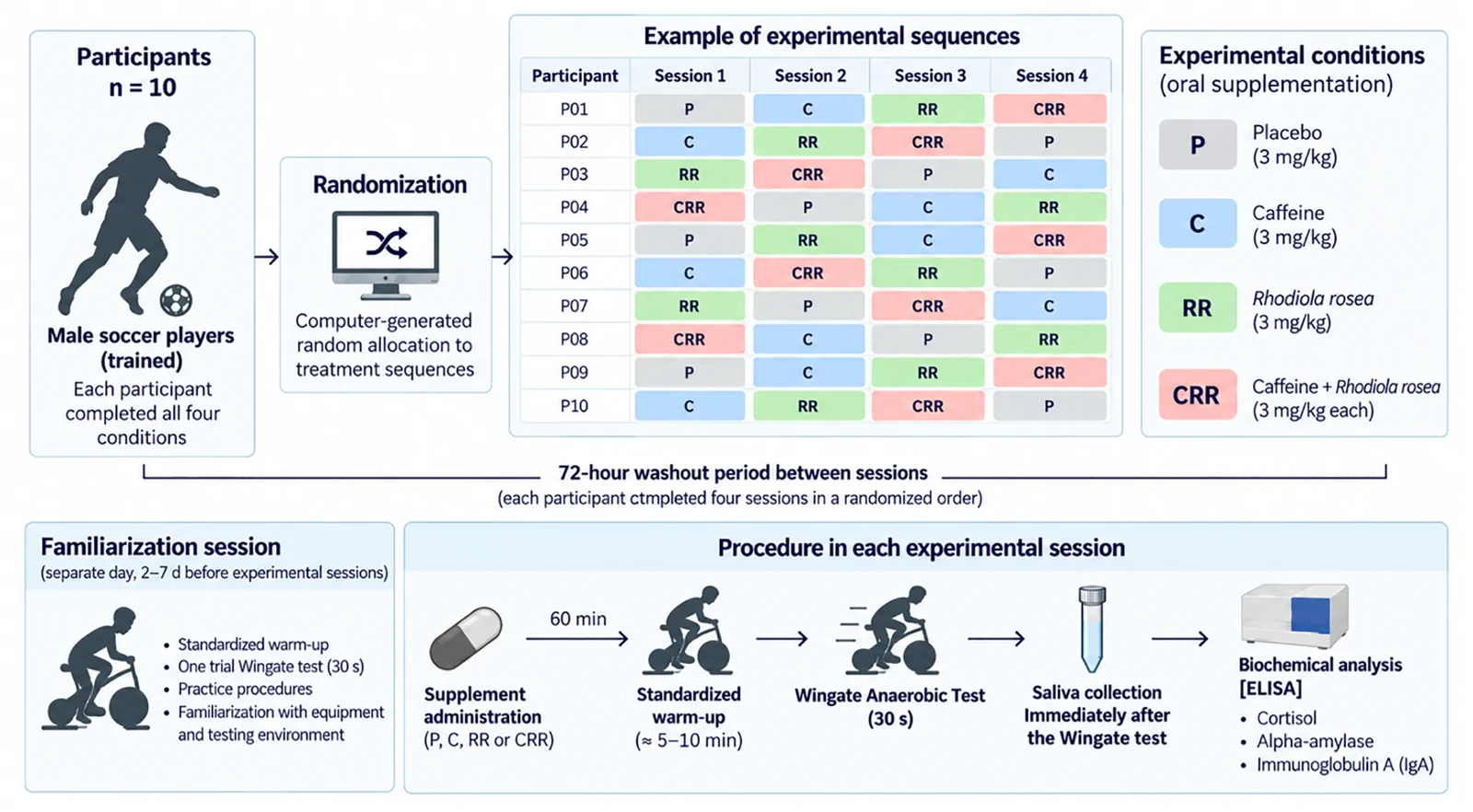
Randomized crossover study design.

### 2.3 Study outcomes

#### 2.3.1 Anthropometric measurements

In the study, body measurements were taken during the first week prior to the implementation of the experimental sessions at 8:00 a.m., on an empty stomach, barefoot, and wearing only shorts, using a scale accurate to ±20 g. Height measurements were taken with a sliding caliper while the students stood upright. The scale was adjusted so that its ends touched the top of the calipers, and height was recorded with a precision of ±1 mm (Louer et al., 2017).

#### 2.3.2 Wingate anaerobic test protocol

On each test day, the athletes’ heights and body weights were recorded prior to the measurements. Participants completed a standardized 10-minute warm-up protocol consisting of a 5-minute light-paced run and 5 minutes of dynamic stretching exercises on each test day. To assess anaerobic performance, the Wingate Anaerobic Test was administered on a Monark 834E mechanical bicycle ergometer (Monark Exercise AB, Vansbro, Sweden). Prior to the test, saddle and handlebar adjustments were individually tailored to each participant’s anthropometric characteristics. The ergometer resistance was set at 7.5% of each participant’s body mass, in accordance with the traditional Wingate Anaerobic Test protocol (Bar-Or, 1987). During the test, participants received standard verbal encouragement to exert maximum effort. The Wingate test was conducted based on the principle of maximum pedaling for 30 seconds and was automatically terminated at the end of the duration. Anaerobic power (peak power) and anaerobic capacity (average power) values were calculated in Watts (W) and relative values in Watts per kilogram (W·kg^-1^) using the Monark Anaerobic Test Software (Version 3) and recorded for analysis (Bar-Or, 1987).

#### 2.3.3 Saliva sampling procedure

Saliva samples were collected in this study to assess biomarkers associated with physiological stress and immune function, including cortisol, α-amylase, and immunoglobulin A (IgA). Saliva samples were collected from all participants under standardized conditions. Prior to each sampling session, participants were instructed not to consume food or beverages, brush their teeth, or chew gum for at least 30 minutes. To minimize the potential influence of circadian variation, all experimental sessions were conducted within the same time window of the day (Granger et al., 2012). In each experimental session, saliva samples were collected immediately after completion of the 30-s Wingate anaerobic test to assess the acute physiological response to the experimental supplementation and exercise protocol. The same sampling procedure and timing were applied consistently across all four experimental conditions to minimize temporal variability in salivary biomarker responses. Saliva was collected using the unstimulated drool method, whereby participants were asked to allow saliva to accumulate naturally in the mouth and then deposit it into sterile collection tubes. Samples with visible intraoral bleeding or other contamination were excluded from the analyses. Following collection, samples were immediately labeled and stored at −20 °C until biochemical analysis. The collection and storage procedures were performed in accordance with previously reported recommendations for maintaining the stability and reliability of salivary biomarkers (Gröschl, 2008).

#### 2.3.4 ELISA analysis procedure

The concentrations of salivary cortisol, α-amylase, and secretory immunoglobulin A (sIgA) were quantitatively determined using enzyme-linked immunosorbent assay (ELISA) (Jaedicke et al., 2012). Saliva samples stored at −20 °C were thawed at room temperature and centrifuged at 1500 × g for 10–15 min to remove particulate material. The resulting supernatants were collected and used for subsequent analyses (Lequin, 2005). Commercially available ELISA kits were used for the determination of salivary cortisol, α-amylase, and sIgA concentrations, following the manufacturer’s instructions (YL Biont, Shanghai, China; cortisol: YLA0549HU; α-amylase: YLA0819HU; sIgA: YLA0762HU). All samples, standards, and controls were analyzed in duplicate. Laboratory personnel performing the analyses were blinded to the experimental conditions to minimize potential measurement bias. For each assay, standard curves were generated using the serially diluted standards provided with the respective kits. Following the prescribed incubation and washing procedures, the tetramethylbenzidine (TMB) substrate was added, and the enzymatic reaction was terminated with the provided stop solution. Optical density was measured at 450 nm using a microplate reader. The concentrations of cortisol, α-amylase, and sIgA were calculated from the corresponding standard curves. According to the manufacturer’s specifications, the intra-assay coefficients of variation were <8% and the inter-assay coefficients of variation were <10% for all three assays. The analytical sensitivity and measurement range were 0.251 ng/mL and 0.5–100 ng/mL for cortisol, 10.13 U/L and 20–6000 U/L for α-amylase, and 0.01 μg/mL and 0.02–7 μg/mL for sIgA, respectively.

#### 2.3.5 Supplementation protocol

##### 2.3.5.1 Rhodiola rosea

Rhodiola rosea (RR) supplementation was provided using a commercially available standardized product (Solgar Rhodiola Root Extract, Solgar Inc., USA). Each vegetable capsule contained 250 mg of standardized RR root extract, providing 3 mg of salidroside (1%) and 8 mg of rosavins (3%), together with 100 mg of non-standardized Rhodiola root powder, according to the manufacturer’s specifications. The total RR extract dose was standardized to 3 mg·kg^-1^ body mass for each participant based on previous research investigating the potential ergogenic effects of RR supplementation (Noreen et al., 2013; Tinsley et al., 2024).

##### 2.3.5.2 Caffeine

Caffeine supplementation was administered using a commercially available product (One Up Caffeine Plus, Dikey Vitamin Kozmetik ve Gıda Takviyeleri A.Ş., Türkiye). The caffeine dose was standardized to 3 mg·kg^-1^ for each participant, consistent with previously reported acute ergogenic protocols (Duncan et al., 2016).

##### 2.3.5.3 Combined caffeine + *Rhodiola rosea*

In the combined caffeine + Rhodiola rosea (CRR) condition, participants simultaneously received 3 mg·kg^-1^ caffeine and 3 mg·kg^-1^ Rhodiola rosea, with each supplement administered in a separate capsule.

##### 2.3.5.4 Placebo and blinding

The placebo capsules contained microcrystalline cellulose and were prepared to be visually identical to the active supplementation capsules in terms of appearance, color, and volume. All capsules were prepared by the research team under identical conditions to ensure consistency across experiments. Participants were blinded to the supplementation condition and did not know which supplement was administered, whereas the investigator responsible for administering supplements was aware of the assigned experimental condition. After each experimental session, participants were asked which supplementation condition they believed they had received; however, these responses were not systematically recorded or analyzed. The visual similarity of the experimental capsules is presented in **Figure 2**.

**Figure 2:**
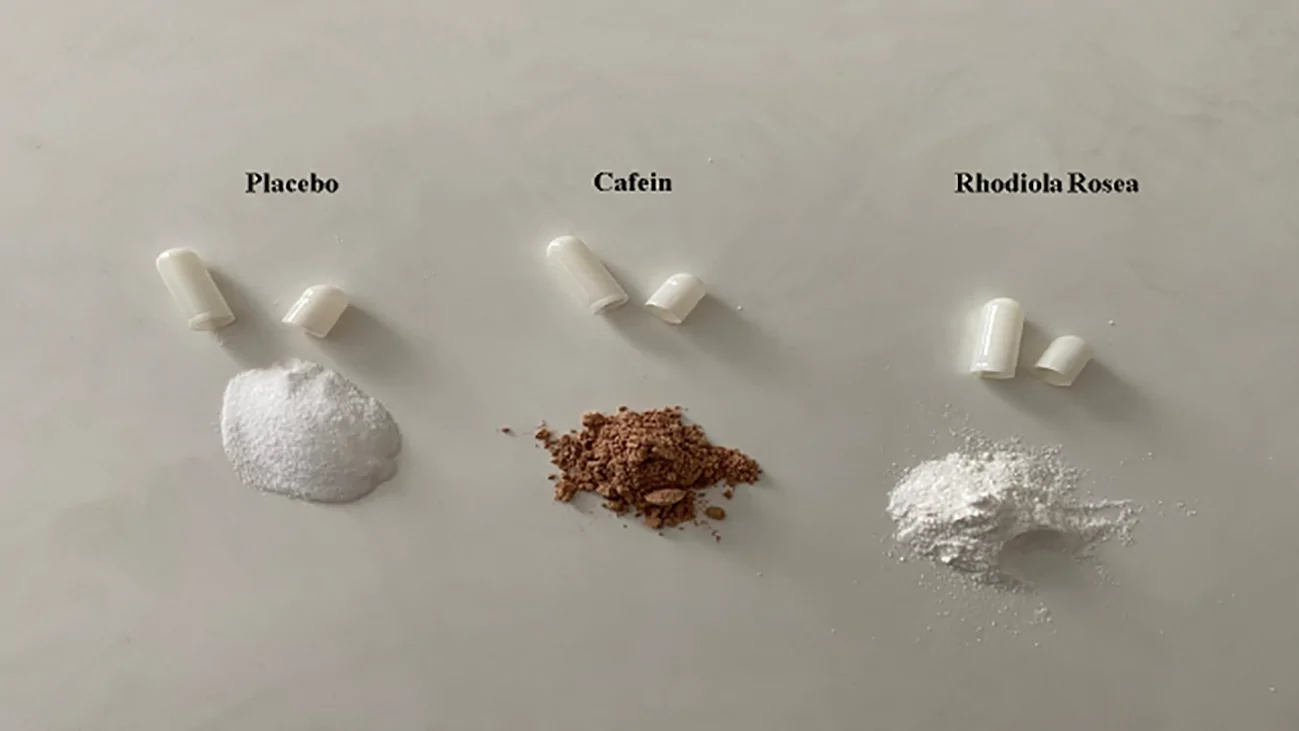
Visual similarity of application content. The CRR condition consisted of the simultaneous administration of the caffeine and Rhodiola rosea capsules shown in the figure and therefore did not involve a separate capsule formulation.

##### 2.3.5.5 Pre-experimental standardization

To minimize potential confounding factors, participants were instructed to abstain from coffee, caffeine-containing products, alcohol, and strenuous physical activity for 72 h before each experimental session. Participants were also instructed to maintain their usual dietary habits throughout the study period. Dietary records were obtained for the 72-h period preceding the first experimental session. All experimental sessions were scheduled at the same time of day for each participant. Participants were also instructed to maintain similar sleep and dietary patterns before each experimental session.

### 2.4 Statistical analysis

The data were analyzed using SPSS 25.0 (IBM, Armonk, NY, USA). Descriptive statistics were calculated, and the assumption of normality was assessed using skewness and kurtosis values (± 2) (George and Mallery, 2019). Since the study employed a single-factor repeated-measures design, a one-way repeated-measures ANOVA was used to assess differences between the four experimental conditions. Treatment/condition was therefore included as the within-subject factor in the primary analysis. When a significant main effect was detected, the Bonferroni *post-hoc* test was used. Effect size was calculated using partial eta-squared (ηp²) and interpreted according to Cohen’s classification (small = 0.01; medium = 0.06; large ≥ 0.14) (Lakens, 2013). Partial eta-squared (ηp²) was selected as the primary effect size measure because it is widely recommended for repeated-measures ANOVA designs and provides an appropriate estimate of experimental condition effects in within-subject crossover studies. The level of statistical significance was set at p < 0.05. The graphs were prepared using GraphPad Prism 8 software. Because of the crossover design, the order of the four experimental conditions was randomized for each participant using the randomization function of Random.org (Randomness and Integrity Services Ltd., Dublin, Ireland). In addition, a 72-h washout period was implemented between experimental sessions to minimize the possibility of residual effects from the preceding condition. Period and sequence effects were not modeled as separate statistical terms in the primary analysis, and a linear mixed-effects model was not used. These potential sources of bias were therefore addressed primarily through the randomized crossover design and the predefined washout interval.

## 3 Results

**Table 1** shows the mean and standard deviation values for anaerobic performance and salivary biomarker results across the four experimental conditions. Maximum power and relative maximum power were highest under the CRR condition. Mean power and relative mean power also reached their highest values under the CRR condition. When salivary biomarkers were examined, cortisol levels reached their highest mean values under the CRR condition, while IgA levels reached their highest mean values under the RR condition; α-amylase levels were highest under the placebo condition. However, statistically significant differences were observed only in the relevant pairwise comparisons. Maximum power was lower in the CRR condition compared to the placebo condition; average power was lower in the CRR condition compared to the RR condition; cortisol was lower in the CRR condition compared to the placebo condition; α-amylase was lower in the caffeine condition compared to the placebo condition; and IgA was higher in the RR condition compared to the placebo condition.

**Table 1:** Descriptive statistics and 95% confidence intervals for anaerobic performance and salivary biomarker outcomes across experimental conditions.

Outcome		Placebo	Caffeine	RR	CRR
Max power (W)	Mean ± SD	755.7 ± 86.47	793.9 ± 138.2	766.1 ± 97.02	867.7 ± 89.31
	95% CI	693.9-817.6	695-892.8	696.7-835.6	803.8-931.6
Relative max power (W/kg)	Mean ± SD	10.91 ± 1.07	11.55 ± 1.81	11.18 ± 1.47	12.12 ± 1.21
	95% CI	10.19-11.63	10.25-12.84	10.13-12.23	11.25-12.99
Mean power (W)	Mean ± SD	551.9 ± 52.99	586.9 ± 63.56	549.4 ± 50.08	599 ± 38.98
	95% CI	514-589.8	541.4-632.4	513.6-585.2	571.1-626.8
Relative mean power (W/kg)	Mean ± SD	8.04 ± 0.59	8.24 ± 0.40	7.99 ± 0.56	8.42 ± 0.37
	95% CI	7.61-8.46	7.96-8.53	7.59-8.40	8.15-8.70
Cortisol	Mean ± SD	52.29 ± 8.23	55.08 ± 7.95	65.14 ± 11.47	66.83 ± 13.27
	95% CI	40.64-58.18	49.39-60.77	56.93-73.34	57.34-76.32
alpha-amylase	Mean ± SD	2553 ± 764.1	1804 ± 285.9	2123 ± 419.2	2084 ± 651.9
	95% CI	2006-3099	1600-2009	1823-2423	1618-2550
IgA	Mean ± SD	94.48 ± 20.38	97.47 ± 32.76	121.9± 30.42	120.3 ± 44.51
	95% CI	79.90-109.1	74.04-120.09	100.1-143.6	88.42-152.1

CI, Confidence Interval.

**Figure 3A** one-way repeated-measures ANOVA with Greenhouse–Geisser correction revealed a significant experimental condition effect on cortisol levels (F(2.054, 18.49)=5.732, p=0.011, ϵ=0.685, ηp²=0.39). Bonferroni-adjusted pairwise comparisons demonstrated significantly higher cortisol values in the CRR condition than in the placebo condition (mean difference = 14.54, 95% CI: 2.47 to 26.60, p = 0.033), whereas no other pairwise comparisons reached statistical significance. **Figure 3B**. A one-way repeated-measures ANOVA with Greenhouse–Geisser correction revealed a significant experimental condition effect on salivary α-amylase levels (F(2.888, 24.57) = 4.443, p = 0.015, ϵ = 0.910, ηp² = 0.33). Pairwise comparisons with Bonferroni correction showed demonstrated significantly lower alpha-amylase values in condition C compared to the placebo condition (mean difference = 748.7; 95% CI: 40.91 to 1538; p = 0.033), whereas no other pairwise comparison reached statistical significance. **Figure 3C** A one-way repeated-measures ANOVA with Greenhouse–Geisser correction revealed a significant experimental condition effect on salivary IgA levels (F(2.215, 19.93) = 3.415, *p* = 0.049, ϵ = 0.738, ηp² = 0.28). Bonferroni-adjusted pairwise comparisons demonstrated significantly higher salivary IgA levels in the RR condition than in the placebo condition (mean difference = 27.37, 95% CI: 5.73 to 49.02*, p =* 0.033). No significant differences were observed between the remaining experimental condition conditions.

**Figure 3:**
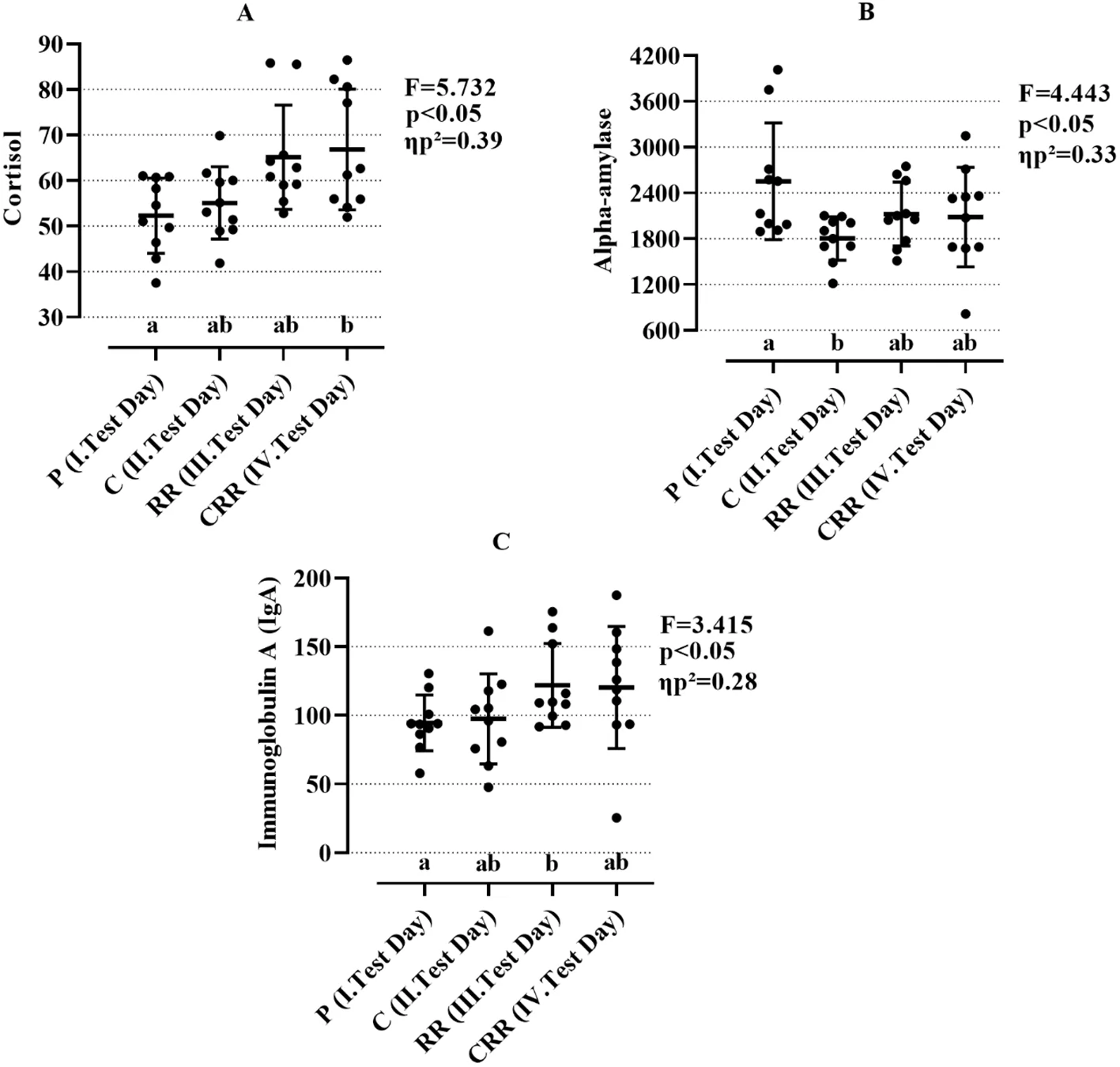
Analysis results for salivary markers. P, Placebo; C, Caffeine; RR, Rhodiola rosea; CRR, Caffeine-Rhodiola rosea. There are statistically significant differences between measurements marked with different letters (p < 0.05). **(A)** Cortisol levels; **(B)** Alpha-amylase levels; **(C)** Immunoglobulin (IgA) levels.

**Figure 4A**. A one-way repeated-measures ANOVA with Greenhouse–Geisser correction revealed a significant experimental condition effect on peak power output (F(1.511, 13.60) = 5.446, *p* = 0.025, ϵ = 0.504, ηp² = 0.38). Bonferroni-adjusted pairwise comparisons demonstrated significantly higher peak power values in the CRR condition than in the placebo condition (mean difference **=** 111.9 W, 95% CI: 1.51 to 222.4 W, *p=*0.023). No significant differences were observed between the remaining experimental condition conditions. **Figure 4B**. A one-way repeated-measures ANOVA with Greenhouse–Geisser correction revealed a significant experimental condition effect on relative peak power output (F(1.959, 17.63) **=** 3.904, *p* = 0.040, ϵ = 0.663, ηp² = 0.30). Bonferroni-adjusted pairwise comparisons demonstrated significantly higher relative peak power values in the CRR condition than in the placebo condition (mean difference **=** 1.212 W/kg, 95% CI: 0.006 to 2.418 W/kg, *p=*0.038). No significant differences were observed between the remaining experimental condition conditions. **Figure 4C**. A one-way repeated-measures ANOVA with Greenhouse–Geisser correction revealed a significant experimental condition effect on mean power output (F(2.120, 19.08) = 6.341, *p* = 0.007, ϵ = 0.706, ηp² = 0.41). Bonferroni-adjusted pairwise comparisons demonstrated significantly higher mean power values in the CRR condition than in the RR condition (mean difference **=** 49.57 W, 95% CI: 0.484 to 98.65 W, *p=*0.040). No significant differences were observed between the remaining experimental condition conditions. **Figure 4D**. Shows that the Average Power (Watt/kg) variable did not vary significantly across the supplements taken by the participants (F(1.837, 16.53) = 3.506, p>0.05, ϵ = 0.612, ηp² = 0.28).

**Figure 4:**
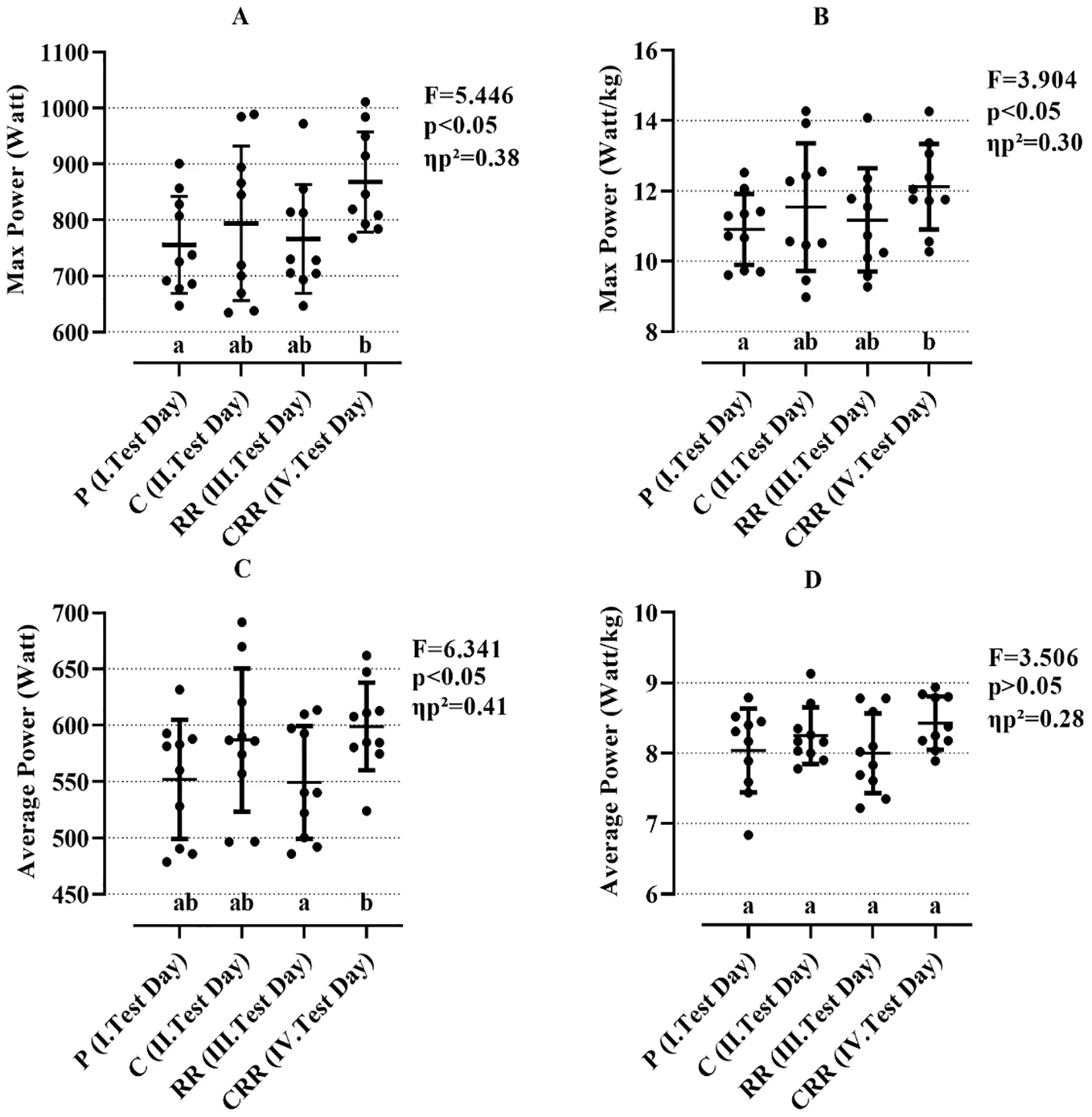
Analysis results for Wingate power parameters. P, Placebo; C, Caffeine; RR, Rhodiola rosea; CRR, Caffeine- Rhodiola rosea. There are statistically significant differences between measurements marked with different letters (p < 0.05). **(A)** Peak power output; **(B)** Relative peak power output; **(C)** Mean power output; **(D)** Avarege power output.

## 4 Discussion

Ergogenic support strategies aimed at enhancing exercise performance are considered a significant area of research in the sports science literature. Caffeine, which is thought to influence central nervous system activation, the regulation of fatigue perception, and the physiological stress response, and RR, known for its adaptogenic properties, are regarded as supplements with the potential to support athletic performance and physiological adaptation. Studies examining the combined effects of these two supplements on neuroendocrine stress responses and salivary stress biomarkers during high-intensity anaerobic exercise remain limited. In this context, the present study investigated the acute effects of caffeine and RR supplementation, administered individually and in combination, on anaerobic performance and salivary biomarkers reflecting the physiological stress response (cortisol, α-amylase, and IgA) in soccer players. The findings indicate that the combined supplementation protocol was associated with improvements in several anaerobic performance parameters and alterations in exercise-induced neuroendocrine stress biomarkers. These findings indicate that the combined administration of caffeine and RR was associated with differences in several anaerobic performance parameters compared with the other experimental conditions. However, as formal interaction analyses were not performed, these findings should not be interpreted as evidence of a combined effect. Rather, they provide preliminary evidence of condition-specific acute responses that require confirmation in larger, well-powered studies.

Findings regarding caffeine use are partially consistent with the literature; some studies report that acute caffeine intake at a dose of 3 mg/kg does not have a significant effect on anaerobic performance (Del Coso et al., 2013; Portillo et al., 2017). This suggests that the effects of caffeine may vary depending on individual tolerance, habituation level, and exercise protocol. Mechanistically, caffeine is known to reduce the perception of fatigue and enhance neuromuscular activation through antagonism of adenosine receptors, thereby increasing central nervous system stimulation. Findings regarding the combined use of RR and caffeine are more variable; while some studies report limited effects (Koozehchian et al., 2025), others indicate that this combination may enhance anaerobic power and explosive performance (Tao et al., 2025; Liu et al., 2023). In the present study, the differences observed in the CRR condition may reflect possible complementary physiological actions of RR and caffeine rather than a confirmed combined interaction. Specifically, the proposed effects of RR on cellular energy metabolism, together with the stimulatory effects of caffeine on the central nervous system, may represent possible explanations for the observed differences in anaerobic performance; however, these mechanisms were not directly evaluated in the present study and therefore remain speculative (Ballmann et al., 2019). Similarly, the differences in post-exercise cortisol concentrations observed between the RR and, particularly, the CRR conditions may be related to the proposed effects of RR on physiological stress regulation, including mechanisms that have been suggested to involve the hypothalamic–pituitary–adrenal (HPA) axis (Panossian and Wikman, 2010). However, because saliva samples in the present study were collected within approximately 1 minute after completion of the Wingate test, these measurements should be interpreted as an early post-exercise cortisol response rather than as an indication of the peak cortisol response or the complete temporal profile of the neuroendocrine stress response. Accordingly, the observed differences should be interpreted cautiously, and the present findings do not establish that RR or the RR–caffeine combination directly modulated HPA-axis activity. Cortisol is involved in the physiological response to acute exercise and contributes to the regulation of energy metabolism during exercise (Hill et al., 2008). Thus, the observed early post-exercise differences in cortisol should not necessarily be interpreted as an adverse stress response, particularly because transient cortisol changes may form part of the normal physiological response to high-intensity exercise. Although chronically elevated cortisol concentrations have been associated with adverse effects on performance and recovery (Hackney, 2006), the present study assessed only an early acute post-exercise time point and therefore does not allow conclusions regarding peak cortisol responses, maladaptive stress responses, or chronic alterations in HPA-axis function. Although previous studies have reported that RR may reduce stress and fatigue (Cropley et al., 2015; Olsson et al., 2009), others have not observed significant hormonal changes following supplementation (Timpmann et al., 2018). These discrepancies suggest that the physiological effects of RR are likely influenced by factors such as dosage, supplementation duration, participant characteristics, and exercise protocol. Accordingly, the present findings indicate that the combined administration of RR and caffeine was associated with differences in certain performance and salivary biomarker outcomes compared with the other experimental conditions. However, these observations should not be interpreted as evidence of a true combined effect, as formal interaction analyses were not performed. Although significant improvements were observed in absolute mean power, similar findings were not evident for relative mean power. This discrepancy may be explained by the normalization of power output to body mass. Absolute mean power reflects the total mechanical work produced, whereas relative mean power accounts for inter-individual differences in body mass. Consequently, improvements in absolute power may become less pronounced after normalization, particularly in studies with relatively homogeneous but not identical body mass characteristics. Therefore, the present findings indicate that the observed between-condition difference was evident for absolute mean power but not for body mass-adjusted mean power (Grgic, 2018).

When alpha-amylase changes were evaluated, the significant difference observed between the caffeine and placebo groups may be consistent with the proposed stimulatory effects of caffeine on sympathetic nervous system activity. Alpha-amylase is widely recognized as a non-invasive marker of sympathoadrenal activity and is commonly used to assess the acute stress response (Granger et al., 2007; Nater and Rohleder, 2009). In this context, receptor antagonism has been proposed as one possible mechanism underlying changes in salivary alpha-amylase, potentially through increased sympathetic and catecholaminergic activity (Graham, 2001). Increased sympathetic activity may increase arousal during exercise by supporting performance-related processes such as heart rate, attentional state, and neuromuscular responses. However, findings regarding the effects of caffeine on cortisol and salivary alpha-amylase have been inconsistent across studies. This suggests that the physiological effects of caffeine may vary depending on factors such as dose, individual caffeine habits, genetic susceptibility, and exercise intensity. Therefore, the present findings may be consistent with caffeine-related changes in sympathetic activity during acute exercise, although this interpretation should be considered cautiously.

When salivary α-amylase changes were evaluated, a significant difference was observed between the caffeine and placebo conditions, with lower α-amylase concentrations in the caffeine condition. Salivary α-amylase is widely recognized as a non-invasive marker associated with sympathetic nervous system activity and is commonly used in the assessment of acute physiological stress responses (Nater and Rohleder, 2009). Although caffeine has been proposed to influence sympathetic and catecholaminergic activity through adenosine receptor antagonism (Graham, 2001), the present study did not directly assess sympathetic activity or catecholamine concentrations. Therefore, the lower α-amylase concentration observed following caffeine supplementation cannot be directly attributed to enhanced sympathetic or catecholaminergic activity. Moreover, previous findings regarding the effects of caffeine on salivary α-amylase have been inconsistent across studies. Such variability may be related to differences in caffeine dose, habitual caffeine consumption, individual characteristics, exercise intensity, and the timing of saliva collection. Accordingly, the lower α-amylase concentration observed in the caffeine condition should be interpreted cautiously and should not be considered direct evidence of altered sympathetic activity. Future studies incorporating repeated salivary measurements together with direct assessments of sympathetic and catecholaminergic activity may help clarify the mechanisms underlying caffeine-related changes in salivary α-amylase.

The significant difference observed between the RR and placebo conditions in post-exercise salivary IgA indicates a between-condition difference in this biomarker. However, this finding should be interpreted cautiously because only post-exercise IgA concentrations were assessed and salivary flow rate was not measured. Therefore, the present findings do not allow conclusions regarding changes in mucosal immune function. IgA is one of the key components of the first line of defense in the mucosal immune system and is regarded as a biomarker sensitive to exercise and stress (Gleeson, 2007). Decreases in IgA levels during intense and repetitive training regimens have been associated with an increased risk of upper respiratory tract infections (Walsh et al., 2011). Therefore, maintaining IgA levels is considered important for preserving mucosal immune function in athletes. The antioxidant and adaptogenic properties of RR have been proposed to influence immune-related responses by modulating inflammatory and neuroendocrine processes (Ballmann et al., 2019). However, these proposed mechanisms were not directly assessed in the present study. Therefore, the observed difference in post-exercise salivary IgA should be interpreted as an acute between-condition difference in salivary IgA concentration rather than evidence of altered mucosal immune function. Further studies incorporating pre- and post-exercise IgA measurements and salivary flow rate assessments are warranted to clarify the potential effects of RR on mucosal immune responses.

A review of the literature generally indicates that the effects of RR and caffeine supplementation on both performance and physiological stress biomarkers vary depending on numerous factors, such as duration of use, dosage, type of exercise, and participant characteristics. Similarly, recent evidence has shown that nutritional supplementation may influence oxidative stress, inflammatory responses, and recovery-related biomarkers following high-intensity exercise, emphasizing the importance of integrating physiological biomarkers with performance outcomes when evaluating ergogenic interventions (Kaçar et al., 2026). This situation demonstrates that the effects of these ergogenic aids are context-specific and cannot be explained by a single, unidirectional mechanism of action. The findings of the present study indicate that the combined administration of RR and caffeine was associated with differences in certain performance and physiological stress-related parameters compared with the other experimental conditions. However, these observations should not be interpreted as evidence of a true combined interaction, as formal interaction analyses were not performed. The concurrent differences observed in anaerobic performance and salivary biomarkers indicate that the experimental conditions elicited distinct acute responses; however, the physiological mechanisms underlying these responses cannot be determined from the present measurements. Caffeine-induced increases in central nervous system activation may enhance neuromuscular recruitment during maximal exercise, whereas the adaptogenic properties of RR have been proposed to support physiological adaptation during high-intensity exercise. These proposed complementary physiological actions may explain the between-condition differences observed in the CRR condition; however, this interpretation remains speculative because the underlying mechanisms were not directly assessed in the present study (Jiménez et al., 2021). Nevertheless, the physiological mechanisms underlying these observations were not directly assessed in the present study and therefore require further investigation. In this regard, the present study contributes to the growing body of literature by evaluating both performance outcomes and physiological stress biomarkers following combined supplementation. From a practical perspective, the present findings provide preliminary information on the acute responses to caffeine and RR supplementation during a laboratory-based anaerobic performance test. However, the extent to which these findings translate to sport-specific performance during training or competitive matches remains uncertain. The observed differences in anaerobic performance and salivary biomarkers may provide a basis for further investigation of the acute physiological responses to combined supplementation. However, because the present study evaluated only the acute effects of supplementation in a relatively small sample of male soccer players, these findings should be considered preliminary. Larger randomized controlled trials including female and male athletes, sport-specific performance measures, and longer-term supplementation protocols are needed before definitive practical recommendations can be made (Mielgo-Ayuso et al., 2019).

## 5 Limitations

Certain limitations should be considered when interpreting the findings of this study. First, the relatively small sample size (n = 10), despite being supported by an *a priori* power analysis and the crossover design, may limit the statistical power to detect smaller effects and reduce the generalizability of the findings. Another limitation of the present study is that baseline salivary biomarker measurements were not obtained prior to supplementation at each experimental session. Therefore, the observed biomarker responses reflect post-exercise values only, and individual changes from pre-supplementation baseline could not be determined. Consequently, it cannot be established with certainty whether the observed post-exercise differences were attributable to the experimental interventions or were partly influenced by normal day-to-day within-subject variability in salivary cortisol, α-amylase, and sIgA. Future studies should obtain pre-supplementation baseline samples at each experimental session to enable within-condition changes from baseline to be assessed and to strengthen the interpretation of intervention-related biomarker responses.

Another limitation is that habitual daily caffeine intake was not quantitatively assessed before the study. Although participants were instructed to abstain from coffee, caffeine-containing products, and other caffeine sources for 72 h before each experimental session, individual differences in habitual caffeine consumption could not be quantified and therefore could not be considered when interpreting individual responses to caffeine supplementation. Future studies should assess habitual caffeine intake quantitatively before the experimental period to better account for inter-individual differences in caffeine responsiveness.

A further methodological limitation is that period and sequence effects were not modeled as separate statistical terms, and a linear mixed-effects model was not used. Although the order of the experimental conditions was randomized and a 72-h washout period was implemented to minimize potential order and residual effects, these design features do not statistically test or eliminate period and sequence effects. Future crossover studies may benefit from statistical models that explicitly account for treatment, period, and sequence effects.

The fact that only acute effects were examined limits the ability to draw conclusions regarding the chronic use of RR and caffeine. Additionally, the fact that the sample consisted solely of male soccer players may limit the generalizability of the findings. Furthermore, the use of only salivary biomarkers in the assessment of physiological stress parameters, without the inclusion of blood-based measurements, is another significant limitation. Salivary flow rate was not measured during sample collection; therefore, sIgA was analyzed strictly as an absolute concentration rather than a secretion rate. This limits the ability to account for potential variations in saliva volume or hydration status that may influence concentration metrics.

Another limitation concerns the timing of saliva collection. Saliva samples were obtained within approximately 1 minute after completion of the Wingate test; therefore, this sampling point may not capture the delayed peak of the salivary cortisol response following acute physiological stress. Accordingly, the cortisol findings should be interpreted as an early post-exercise response rather than as an indication of the peak neuroendocrine stress response. In addition, the use of a single post-exercise sampling point prevented characterization of the temporal trajectory of cortisol and the other salivary biomarkers. Future studies should incorporate multiple post-exercise sampling points to better characterize the time course of salivary biomarker responses.

A further important limitation concerns the blinding of the experimental conditions. While the P, C, and RR conditions involved the administration of a single capsule, the combined CRR condition involved the simultaneous administration of two separate capsules. Consequently, participants may have been able to distinguish the CRR condition from the other experimental conditions based on the number of capsules administered. Because the CRR condition was also the only condition in which positive findings were observed, the potential contribution of expectancy effects cannot be completely excluded. A double-dummy design, in which all experimental conditions involve the same number of capsules, would have provided more robust blinding across conditions. Moreover, no formal manipulation check of blinding success was conducted in the present study; therefore, the extent to which participants correctly identified or inferred their experimental condition could not be determined. Future studies should employ a double-dummy design and include a formal assessment of blinding success to better distinguish pharmacological effects from potential expectancy-related effects.

Finally, while a sphericity correction of ϵ = 1.00 was assumed in the *a priori* sample size calculation, lower Greenhouse–Geisser epsilon values were observed for various outcomes, indicating deviations from the assumptions used in the original power calculation. Therefore, the predefined 80% power should be interpreted not as evidence of the power achieved under the observed covariance structure, but as the target power under the *a priori* assumptions. In addition, the assumed effect size of f = 0.40 was not derived from a single empirical estimate from a previous study; therefore, the resulting sample size should be interpreted in the context of the prespecified effect-size assumption. These limitations also serve as suggestions for future research.

## 6 Conclusions

This study provides preliminary evidence regarding the acute effects of caffeine and RR supplementation on selected anaerobic performance outcomes and salivary biomarkers in male soccer players. Differences were observed among the experimental conditions in several performance and salivary biomarker parameters; however, these findings should not be interpreted as evidence of a confirmed interaction between caffeine and RR or as evidence that combined supplementation produces superior effects. The findings should be interpreted cautiously given the small sample size, acute study design, male-only sample, and laboratory-based performance assessment. Further studies with larger and more diverse samples, sport-specific performance measures, and appropriate interaction analyses are warranted to clarify the acute and longer-term effects of caffeine and RR supplementation.

## Data Availability

The raw data supporting the conclusions of this article will be made available by the authors, without undue reservation.
